# On‐Demand, Contact‐Less and Loss‐Less Droplet Manipulation via Contact Electrification

**DOI:** 10.1002/advs.202308101

**Published:** 2024-01-17

**Authors:** Wei Wang, Hamed Vahabi, Arsalan Taassob, Sreekiran Pillai, Arun Kumar Kota

**Affiliations:** ^1^ Department of Mechanical and Aerospace Engineering North Carolina State University Raleigh NC 27695 USA; ^2^ Department of Mechanical Aerospace and Biomedical Engineering University of Tennessee Knoxville Knoxville TN 37996 USA; ^3^ Department of Mechanical Engineering Colorado State University Fort Collins CO 80525 USA

**Keywords:** adhesion, contact electrification, droplet manipulation, electrostatic force, slippery surface

## Abstract

While there are many droplet manipulation techniques, all of them suffer from at least one of the following drawbacks – complex fabrication or complex equipment or liquid loss. In this work, a simple and portable technique is demonstrated that enables on‐demand, contact‐less and loss‐less manipulation of liquid droplets through a combination of contact electrification and slipperiness. In conjunction with numerical simulations, a quantitative analysis is presented to explain the onset of droplet motion. Utilizing the contact electrification technique, contact‐less and loss‐less manipulation of polar and non‐polar liquid droplets on different surface chemistries and geometries is demonstrated. It is envisioned that the technique can pave the way to simple, inexpensive, and portable lab on a chip and point of care devices.

## Graphical abstract

A simple and portable droplet manipulation technique is presented that enables on‐demand, contact‐less and loss‐less manipulation of droplets through a combination of contact electrification and fluorocarbon‐free slippery coating. It is envisioned that the technique will pave the way to simple, inexpensive, and portable lab on a chip and point of care devices.

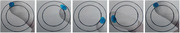

## 1 Introduction

Droplet manipulation refers to actuation of liquid droplets using external stimuli. Droplet manipulation has gained significant interest in recent years because of its applications in microfluidics,^[^ ^1^, ^2^, ^3^ ^]^ chemical synthesis,^[^ ^4^ ^]^ biomedical systems^[^ ^5^, ^6^, ^7^ ^]^ and energy harvesting.^[^ ^8^, ^9^ ^]^ Prior work has demonstrated actuation of droplets using a wide range of stimuli including acoustic waves,^[^ ^10^, ^11^ ^]^ electrical fields,^[^ ^12^ ^]^ light,^[^ ^13^ ^]^ heat,^[^ ^14^ ^]^ magnetic fields^[^ ^15^, ^16^, ^17^ ^]^ etc. However, each of these techniques suffer from at least one of the following drawbacks – complex fabrication or complex equipment or liquid loss. Droplet manipulation based on contact electrification^[^ ^18^, ^19^, ^20^, ^21^ ^]^ (i.e., contact‐induced surface charging) overcomes challenges related to complex fabrication and complex equipment. Droplet manipulation on super‐repellent surfaces^[^ ^22^, ^23^, ^24^, ^25^, ^26^ ^]^ (i.e., surfaces that are extremely repellent and slippery to droplets) overcomes challenges related to liquid loss. So, prior reports demonstrated droplet manipulation by combining contact electrification and superhydrophobic surfaces (i.e., surfaces that are extremely repellent and slippery to water or aqueous liquids).^[^ ^27^, ^28^ ^]^ However, superhydrophobic surfaces are not very durable,^[^ ^29^ ^]^ and can lead to droplet pinning in the Wenzel state,^[^ ^30^ ^]^ especially for low surface tension liquids. While non‐textured, all‐solid slippery surfaces^[^ ^31^ ^]^ can circumvent the concerns related to super‐repellent surfaces, contact electrification‐based droplet manipulation on non‐textured, all‐solid slippery surfaces is very rare.^[^ ^32^ ^]^ In this work, we developed a simple and portable technique that utilizes contact electrification‐based droplet manipulation on fluorocarbon‐free, non‐textured, all‐solid slippery surfaces for on‐demand, contact‐less and loss‐less manipulation of liquid droplets, with both attractive and repulsive electrostatic forces. Furthermore, in conjunction with numerical simulations, we present a quantitative analysis to explain the onset of droplet motion. Utilizing our contact electrification technique, we demonstrate contact‐less and loss‐less manipulation of droplets (polar and non‐polar) with a simple and portable finger‐based actuator (i.e., a wearable device) on different surface chemistries and geometries.

**Figure 1:**
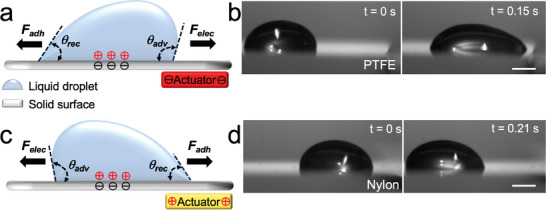
Droplet manipulation with a charged actuator. a) Schematic representing the onset of droplet manipulation with a negatively charged actuator. b) Attraction between a positively charged water droplet and a negatively charged (PTFE) actuator. c) Schematic representing the onset of droplet manipulation with positively charged actuator. d) Repulsion between a positively charged water droplet and a positively charged (Nylon) actuator. Scale bars represent 2 mm.

Our on‐demand, contact‐less and loss‐less droplet manipulation strategy relies on two physical phenomena – contact electrification and slipperiness. Contact electrification is a contact‐induced surface charging phenomenon, wherein surfaces acquire charges of opposite signs (i.e., positive and negative) after contact or friction between surfaces.^[^ ^18^, ^19^, ^20^, ^21^ ^]^ For example, when a droplet is dispensed onto a solid surface, it can acquire charges through contact electrification (**Figure** **1**a,c). The magnitude and sign of the charge acquired by the droplet depend on the solid–liquid interactions.^[^ ^18^, ^19^, ^33^ ^]^ Similarly, upon frictional contact (e.g., simple rubbing) between two solid surfaces, they can acquire positive or negative charges through contact electrification. The magnitude and sign of the charges acquired by the solid surfaces are governed by a triboelectric series.^[^ ^34^, ^35^, ^36^ ^]^ Materials further apart in the triboelectric series, under frictional contact, lead to surface symmetry breakage, thereby gaining or losing electrons. This gain or loss of electrons at the surface induces negative or positive charges, respectively. If such a charged solid surface (an actuator) is brought into the vicinity of a charged droplet (Figure 1a,c), an electrostatic force *F*_*elec*_ = *q_d_E_a_* (as a first order approximation; assuming uniform charge distribution on the droplet) would exist between the actuator and the charged droplet.^[^ ^37^, ^38^, ^39^ ^]^ Here, *q_d_* is the droplet charge and *E_a_* is the electric field intensity between the actuator and the droplet. Depending on the type of charge on the actuator and the droplet, this electrostatic force is either attractive (Figure 1a) or repulsive (Figure 1c). This electrostatic force *F_elec_* between the charged droplet and the actuator can induce motion of the droplet on‐demand, in a contact‐less manner, when it overcomes the lateral adhesion *F*_*adh*_ ≈  *γ*_*lv*_ *D_TCL_* (cos *θ*_*rec*_ − cos *θ*_*adv*_), at the solid–liquid interface.^[^ ^40^, ^41^ ^]^ Here, *γ_lv_* is the liquid surface tension, *D_TCL_* is the width of solid–liquid–vapor contact line perpendicular to the direction of droplet motion, and *θ_adv_* and *θ_rec_* are the advancing and receding contact angles, respectively, of the droplet on the solid surface. Solid surfaces with low contact angle hysteresis (*Δθ* = *θ*_*adv*_ − *θ*_*rec*_) can be slippery and facilitate loss‐less motion of droplets with very low lateral adhesion *F_adh_*. In summary, the onset of such droplet motion occurs when *F*_*elec*_ >  *F*_*adh*_ or when the non‐dimensional force F* = *F_elec_*/*F_adh_* > 1.

## 2 Results and Discussion

In this work, we fabricated slippery solid surfaces with low contact angle hysteresis by modifying smooth glass cover slips with chlorine‐terminated polydimethylsiloxane (Cl‐PDMS) via silanization (see Experimental Section). The low contact angle hysteresis (*θ*_*adv*_ = 103°, *θ*_*rec*_ = 98°, and *Δθ* = 5° for water) on Cl‐PDMS modified glass surfaces arises from the smoothness of the surface and the liquid‐like nature of the flexible siloxane bonds.^[^ ^42^, ^43^, ^44^ ^]^ When a water droplet is dispensed on to the Cl‐PDMS modified glass surface, it acquires a positive charge;^[^ ^27^, ^45^, ^46^, ^47^ ^]^ and the water droplet can be manipulated on‐demand, in a contact‐less and loss‐less manner, by a charged actuator. We prepared charged actuators by simply rubbing nylon and polytetrafluoroethylene (PTFE) sheets against each other. Upon frictional contact, PTFE surfaces acquired negative charges and nylon surfaces acquired positive charges. When such a negatively charged PTFE actuator was brought into the vicinity of the positively charged water droplet on the Cl‐PDMS modified glass surface, the water droplet experienced an attractive force and moved towards the actuator (Figure 1b; Movie S1, Supporting Information); similarly, when a positively charged nylon actuator was brought into the vicinity of the positively charged water droplet on the Cl‐PDMS modified glass surface, the water droplet experienced a repulsive force and moved away from the actuator (Figure 1d; Movie S2, Supporting Information).

**Figure 2:**
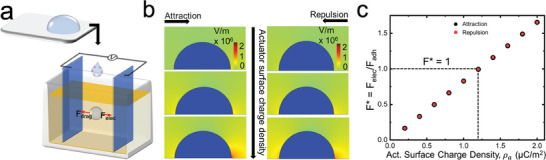
Influence of actuator surface charge density on droplet manipulation. a) Schematic illustrating the measurement of water droplet surface charge density. b) Images showing the non‐uniform distribution of electric field around the water droplet, obtained from numerical simulations. Colors represent the electric field intensity. Electric field intensity increased with increasing actuator surface charge density. c) The non‐dimensional force F* increased with increasing actuator surface charge density. Onset of droplet motion occurred when F* > 1.

To quantitatively understand the onset of droplet motion under attractive or repulsive electrostatic forces, we estimated the lateral adhesion force *F_adh_* at the solid–liquid interface and compared it with the electrostatic force *F_elec_* (tangential to the substrate, in the direction of droplet motion) between the droplet and the actuator. Here, it must be noted that the lateral adhesion and electrostatic forces depend on a wide range of droplet, substrate, actuator, and environmental properties as well as geometric variables. We estimated the lateral adhesion and electrostatic forces while holding all parameters constant except the actuator charge density *ρ*_*a*_. For a 50 µL water droplet on Cl‐PDMS modified glass surface, we estimated the lateral adhesion force *F_adh_* ≈ 32 µN (see Section S1, Supporting Information). Then, to estimate the electrostatic force *F*_*elec*_ between the droplet and the actuator, we conducted a series of systematic experiments and numerical simulations with a fixed water droplet charge *q_d_* and different positive and negative actuator surface charge densities *ρ*_*a*_. We measured the droplet charge *q_d_* using a Millikan droplet apparatus (**Figure** **2**a; see Experimental Section) consisting of two parallel plate electrodes immersed in silicone oil with a uniform electric field between them. A 50 µL water droplet was first dispensed on to the Cl‐PDMS modified glass surface, and after it acquired the positive charge, it was dropped into the silicone oil between the parallel plate electrodes. Without an electric field between the electrodes, the charged droplet descended along a vertical trajectory. Upon applying an electric field between the electrodes, the positively charged droplet experienced an electrostatic force, and was pulled towards the negative electrode, thereby deviating from the vertical trajectory. By tracking the droplet trajectory using a high‐speed camera (see Experimental Section), the droplet charge was estimated from a balance between the electrostatic force and the hydrodynamic force as, *q_d_* = 4π*µ*_*oil*_ *R_d_U_d_d*(3*λ* + 2)/*V*(2*λ* + 2) ≈ 0.18 nC (see Section S2, Supporting Information).^[^ ^37^, ^48^, ^49^ ^]^ Here, *µ*_oil_ is the viscosity of the silicone oil, *U_d_* is the horizontal velocity of the droplet, *R_d_* is the radius of the droplet, and *λ* is the ratio of viscosity of water and silicone oil, *V* is the applied voltage and *d* is the distance between the electrodes. We fabricated actuators with six different surface charge densities *ρ*_*a*_ by rubbing nylon and PTFE sheets against each other for different times. We measured actuator surface charge densities using a surface voltmeter (see Experimental Section). Then, we estimated the electrostatic force *F*_*elec*_ (tangential to the substrate, in the direction of droplet motion) between the actuator and the droplet by numerically solving Gauss's law with a finite element‐based solver in COMSOL (see Experimental Section). As anticipated, with increasing actuator surface charge density, our numerical simulations indicated that the electric field around the droplet increased (Figure 2b), thereby increasing the electrostatic force *F_elec_*. Since the water droplet was positively charged, the negatively charged actuator resulted in an attractive force and the positively charged actuator resulted in a repulsive force. The magnitude of electrostatic force *F_elec_* experienced by the droplet was similar for attractive and repulsive droplet manipulation systems. At low actuator surface charge densities (*ρ*_*a*_<< 1.2 µC m^−2^), our simulations indicated that the non‐dimensional force F* < 1. Correspondingly, in our experiments, the water droplets remained adhered to the surface and did not move because the electrostatic force *F*_*elec*_ could not overcome the lateral adhesion *F_adh_* (Figure 2c). At sufficiently high actuator surface charge densities (*ρ*_*a*_>>1.2 µC m^−2^), our simulations indicated that the non‐dimensional force F*>1. Correspondingly, in our experiments, the water droplets slid past the surface because the electrostatic force *F_elec_* overcame the lateral adhesion *F_adh_*. The slipperiness (due to low contact angle hysteresis) ensured that there was virtually no liquid residue left behind (Section S3, Figure S1a–c, Supporting Information). In this manner, our simple technique utilizes surface charges obtained by contact or friction between surfaces for on‐demand, contact‐less and loss‐less manipulation of water droplets.

**Figure 3:**
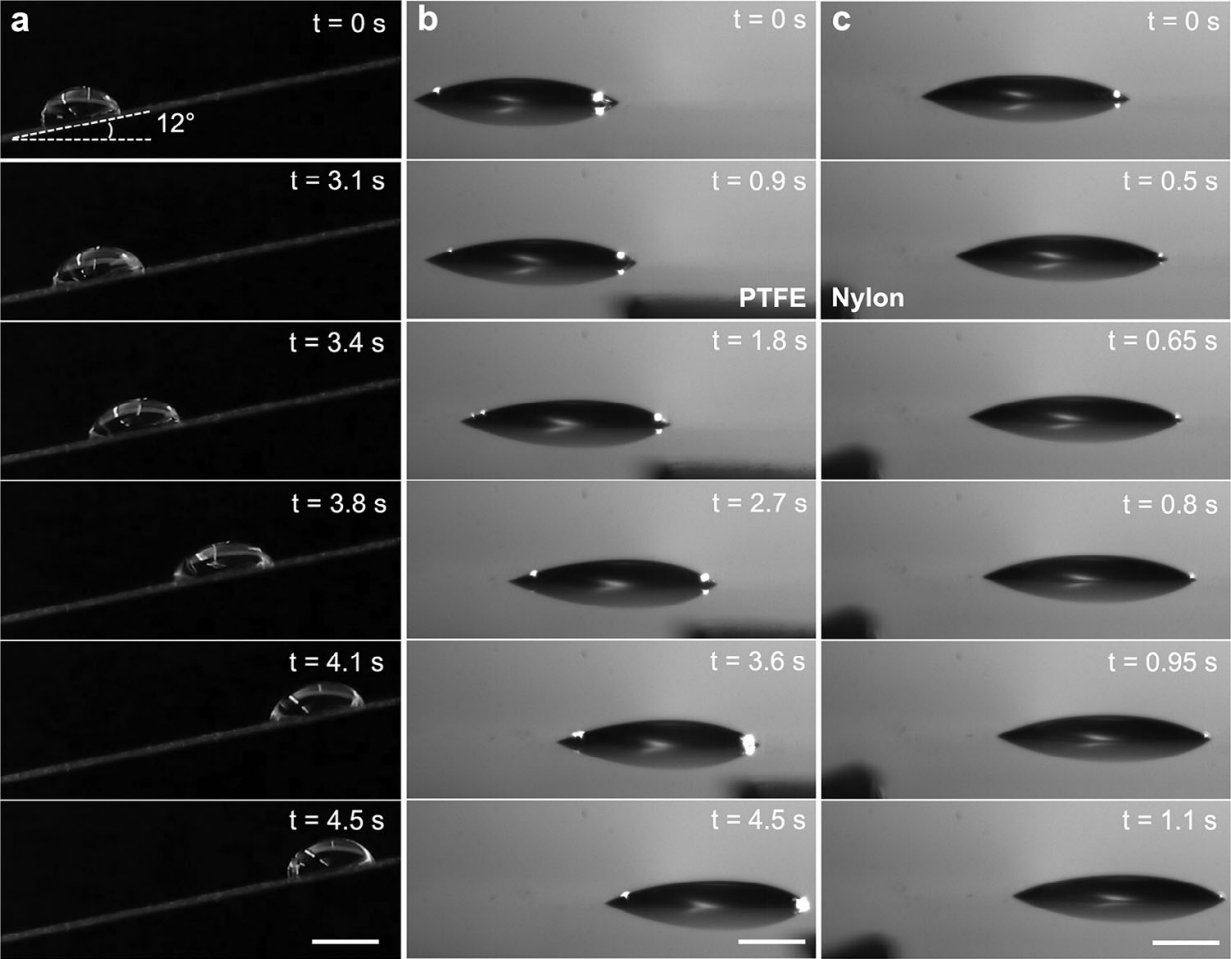
Droplet manipulation with different geometry and chemistry. a) Snapshots showing uphill motion of a water droplet on OTS modified glass surface due to repulsion by a positively charged actuator (Nylon). b) Snapshots showing attraction between hexadecane droplet and a negatively charged (PTFE) actuator on a Cl‐PDMS modified glass surface. c) Snapshots showing repulsion between hexadecane droplet and a positively charged (Nylon) actuator on a Cl‐PDMS modified glass surface. Scale bars represent 5 mm.

Our droplet manipulation technique based on contact electrification is versatile and can be used with different surface chemistries and geometries. To demonstrate this, we fabricated glass surfaces modified with octadecyltricholorsilane (OTS) via silanization (see Experimental Section) and placed them tilted at ≈10° relative to the horizontal. Then, we dispensed a 50 µL water droplet on the OTS‐modified glass surface (*θ*_*adv*_ = 103°, *θ*_*rec*_ = 91°, and *Δθ* = 12° for water), and the water droplet acquired a positive charge upon contact.^[^ ^27^, ^45^, ^46^, ^47^ ^]^ When a positively charged nylon actuator was brought near the lower end of the positively charged water droplet on the OTS modified glass surface, the water droplet experienced a repulsive force and moved uphill, away from the actuator (**Figure** **3**a; Movie S3, Supporting Information). The uphill motion of the droplet indicates that the electrostatic force experienced by the droplet was higher than the sum of the lateral adhesion and the gravitational forces along the surface. With the actuator held stationary at the initial position, the droplet continued moving uphill and eventually stopped after moving sufficiently away from the actuator, indicating that the electrostatic force can no longer overcome the sum of the lateral adhesion and the gravitational forces. In this manner, our simple technique can enable on‐demand, contact‐less and loss‐less manipulation of water droplets on different surface chemistries and geometries.

In addition to polar liquids like water, our technique allows on‐demand, contact‐less and loss‐less manipulation of non‐polar liquids like oils (e.g., hexadecane). Such manipulation of non‐polar liquid droplets is counter‐intuitive because they are considered intrinsically neutral and do not easily accommodate electric charges,^[^ ^50^, ^51^, ^52^ ^]^ and in such cases, one cannot anticipate droplet manipulation using electrostatic forces. However, rather surprisingly, when a droplet of hexadecane was dispensed on to Cl‐PDMS modified glass surface (*θ*_*adv*_ = 42°, *θ*_*rec*_ = 39°, and *Δθ* = 3° for hexadecane), it acquired a positive charge. When a negatively charged PTFE actuator was brought into the vicinity of the hexadecane droplet on Cl‐PDMS modified glass surface, the hexadecane droplet experienced an attractive force and moved towards the actuator (Figure 3b); similarly, when a positively charged nylon actuator was brought into the vicinity of the hexadecane droplet on the Cl‐PDMS modified glass surface, the hexadecane droplet experienced a repulsive force and moved away from the actuator (Figure 3c). This counter‐intuitive manipulation of non‐polar liquids is possibly due to trace amounts of polar impurities (hexadecane used in this work is at least 99.9% pure), which can alter the electrical properties by promoting charge carriers and facilitating conduction.^[^ ^53^, ^54^, ^55^ ^]^ In this manner, our simple technique can allow on‐demand, contact‐less and loss‐less manipulation of non‐polar liquid droplets with trace amounts of impurities (i.e., common commercially available oils), when the electrostatic force overcomes lateral adhesion.

**Figure 4:**
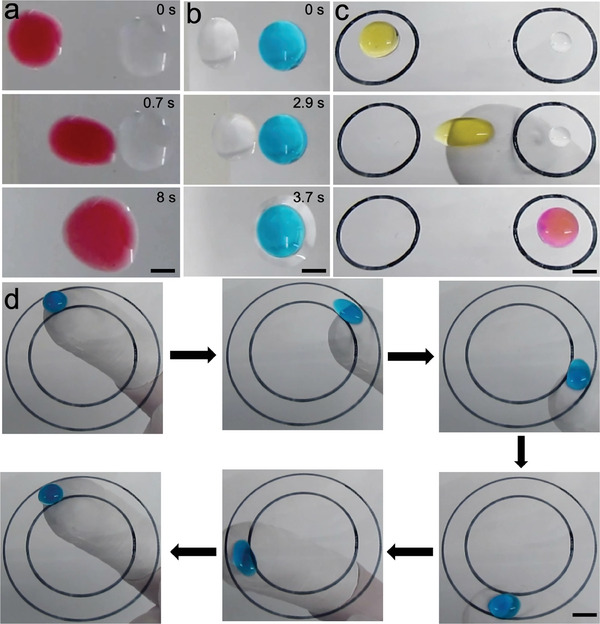
Potential applications of the on‐demand, contact‐less and loss‐less droplet manipulation. Snapshots showing the transportation and merging of a) miscible droplets – hexadecane (colorless) and toluene (red), and b) immiscible droplets – hexadecane (colorless) and water (blue). Scale bar represents 2 mm. c) Snapshots showing colorimetric indication of droplet pH. A flexible PTFE tape wrapped around a finger was used as an actuator to transport and merge a 50 µg mL^−1^ phenol red in water droplet (yellow) with a 0.1 Mm sodium hydroxide droplet (colorless). d) Manipulation of a water droplet (blue) along a circular (arbitrary) path using a finger wrapped with a flexible PTFE tape. In (c) and (d) black circles are drawn underneath the glass surface for better visualization. Scale bars represent 5 mm.

Finally, our simple droplet manipulation technique enables on‐demand, contact‐less and loss‐less transportation and merging of miscible or immiscible liquid droplets, which is desirable for a wide variety of applications. To demonstrate merging of miscible liquids (**Figure** **4**a; Movie S4, Supporting Information), we used droplets of toluene (red, left) and hexadecane (colorless, right). When a positively charged nylon actuator was brought near the left end of the positively charged toluene droplet, the toluene droplet experienced a repulsive force and moved away from the actuator, towards the hexadecane droplet, and the droplets coalesced together. Similarly, to demonstrate merging of immiscible liquids (Figure 4b; Movie S5, Supporting Information), we used droplets of hexadecane (colorless, left) and water (blue, right). When a positively charged nylon actuator was brought near the left end of the positively charged hexadecane droplet, the hexadecane droplet experienced a repulsive force and moved away from the actuator, towards the water droplet, and the droplets coalesced together. Furthermore, our technique can be used for colorimetric indication of droplet pH. To demonstrate colorimetric indication of pH (Figure 4c; Movie S6, Supporting Information), we used droplets of phenol red in water (yellow, left) and sodium hydroxide (colorless, right). For actuation, rather than using rigid actuators, we simply used a flexible PTFE tape wrapped around a finger as an actuator. Simply rubbing the PTFE wrapped finger against nearly any surface, including our own skin, induces negative charges. When such a negatively charged PTFE tape‐wrapped‐finger was brought near the right end of the positively charged droplet of phenol red in water, the droplet experienced an attractive force, and as the finger moved from left to right under the substrate, the droplet slid synchronously, and eventually coalesced with the sodium hydroxide droplet. Subsequently, the coalesced droplet changed color to pink, indicating a pH > 8.2,^[^ ^56^ ^]^ confirming the basic nature of the sodium hydroxide droplet. Such finger‐based actuation enables on‐demand, contact‐less and loss‐less manipulation of droplets along arbitrary paths. To demonstrate such droplet manipulation along an arbitrary path (Figure 4d; Movie S7, Supporting Information), we used a droplet of water (blue). When a negatively charged PTFE tape‐wrapped‐finger was brought near the water droplet, it experienced an attractive force, and as the finger moved along an arbitrary circular path, the droplet slid synchronously. In this manner, our technique enables on‐demand and contact‐less droplet manipulation without complex fabrication or complex equipment or liquid loss.

## 3 Conclusion

In this work, we designed and demonstrated on‐demand, contact‐less and loss‐less manipulation of liquid droplets by combining contact electrification and slipperiness. We presented a quantitative analysis to explain that on‐demand, contact‐less manipulation of liquid droplets occurs when the electrostatic force overcomes the adhesion force, i.e., *F*_*elec*_ >  *F*_*adh*_. Surface slipperiness enables loss‐less manipulation due to the low contact angle hysteresis, and consequently, low lateral adhesion. Building on this understanding, we used negatively‐charged or positively‐charged actuators to manipulate charged liquid droplets via attraction or repulsion. We demonstrated the versatility of our technique by using different surface chemistries and geometries, manipulating polar and non‐polar liquid droplets, as well as merging miscible and immiscible liquid droplets. Finally, we demonstrated the simplicity and portability of technique using a finger‐based actuator to manipulate droplets along arbitrary paths. While a comprehensive study to investigate the influence of droplet, actuator, substrate, and environmental properties as well as geometric variables is needed for a thorough understanding of contact electrification‐based droplet manipulation, we envision that our on‐demand, contact‐less and loss‐less droplet manipulation technique as well as the quantitative analysis will pave the way for novel contact electrification‐based lab on a chip and point of care devices.

## 4 Experimental Section

### Materials

Glass coverslips, glass beakers, sodium hydroxide (≥98% purity), hexadecane (≥99.9% purity), oil Red O (red dye) and food color (blue dye) were purchased from Fisher Scientific. Toluene (≥99.3% purity) and ethanol were purchased from Sigma–Aldrich. Phenol Red pH indicator was purchased from Millipore Sigma. Chlorine terminated polydimethylsiloxane (Cl‐PDMS, molecular weight ≈ 425–650 Da, viscosity ≈ 3–8 cSt), octadecyltrichlorosilane (OTS) and silicone oil (polydimethylsiloxane, trimethylsiloxy terminated, viscosity ≈ 20 cSt) were purchased from Gelest. Copper sheets, PTFE sheets, nylon sheets and PTFE tape were purchased from McMaster. DI water was obtained from a water purification system (Sartorius Arium Mini).

### Fabrication of Slippery Surfaces

Glass coverslips were cleaned by rinsing thoroughly with ethanol and DI water, and then dried with nitrogen. The cleaned substrates were exposed to oxygen plasma (PlasmaEtch PE‐25) at a pressure of 200 mTorr for 15 min for hydroxylation. To prepare Cl‐PDMS modified glass surfaces, the hydroxylated surfaces were exposed to vapors of 150 µL Cl‐PDMS at 150 °C for 60 min. Then, the surfaces were sequentially rinsed with toluene and ethanol, and dried at 70 °C for 120 min. To prepare OTS modified glass surfaces, the hydroxylated substrates were exposed to the vapors of 150 µL OTS at 150 °C for 60 min. Then, the surfaces were sequentially rinsed with toluene, DI water and ethanol, and dried with nitrogen.

### Contact Angles

Contact angles were measured using a goniometer (Ramé‐Hart 260‐F4). The contact angles were measured by advancing or receding a small volume of liquid (≈ 5 µL) onto the surface using a 2 mL micrometer syringe (Gilmont). All results are an average of six individual measurements performed on each surface.

### Actuator Surface Charge Density

First, PTFE and nylon sheets (48 mm × 20 mm) were mounted on glass slides and discharged with an anti‐static gun until surface voltage <0.01 kV. Then, the PTFE and nylon sheets were rubbed against each other (applied force ≈2 N, rub area ≈960 mm^2^) to prepare positively‐charged nylon actuator and negatively‐charged PTFE actuator. Finally, the actuator charge density was measured using a surface DC voltmeter (AlphaLab Inc. SVM2) placed at a distance of ≈30 mm from the actuator. Each measurement was conducted at three spatially different locations and the average was reported as the measured surface charge density.

### Droplet Surface Charge Density

The Millikan droplet apparatus consisted of two parallel copper sheets (electrodes) 74 mm apart, immersed in a glass beaker containing silicone oil. A potential of 8 kV was applied across the electrodes using a DC power source (Gamma High Voltage Research ES‐30P5W). The charged water droplet was dispensed between the electrodes, into the silicone oil, and its trajectory was recorded with a high‐speed camera (Photron Fastcam SA3). The recorded videos were analyzed (ImageJ) to estimate the horizontal terminal velocity of the droplet.

### COMSOL Simulations

The electrostatic force *F*_*elec*_ between the actuator and the charged water droplet was estimated using COMSOL Multiphysics simulations. In the simulation domain, the geometry, material properties and surface charge densities of the actuators were consistent with the experiments. The electric field due to each actuator was obtained by numerically solving Gauss's law *∇* · *E_a_* =  *ρ*_*a*_/*ε*. Here, *∇* · *E_a_* is the divergence of the electric field, *ρ*_*a*_ is the actuator surface charge density, and *ε* is the electric permittivity of the material within the simulation domain. The finite element‐based solver in COMSOL discretizes the geometry and numerically solves for the non‐uniform electric field intensity *E_a_* in the simulation domain. Then, the electrostatic force was estimated as *F*_*elec*_ = *q_d_E_a_* (tangential to the substrate, in the direction of droplet motion or opposite to lateral adhesion), between the actuator and the charged water droplet. Here, *q_d_* is the droplet charge and *E_a_* is the component of electric field between the actuator and the droplet, tangential to the substrate, in the direction of droplet motion.

## Conflict of Interest

The authors declare no conflict of interest.

## Supporting information

Supporting Information

Supplemental Movie 1

Supplemental Movie 2

Supplemental Movie 3

Supplemental Movie 4

Supplemental Movie 5

Supplemental Movie 6

Supplemental Movie 7

## Data Availability

The data that support the findings of this study are available from the corresponding author upon reasonable request.
