# Supplementary material for: On‐Demand, Contact‐Less and Loss‐Less Droplet Manipulation via Contact Electrification
Source: Adv Sci (Weinh). 2024 Jan 17;11(10):2308101. doi: 10.1002/advs.202308101 (PMC10933654; doi:10.1002/advs.202308101)
Supplement: Supplementary file 1 — Supporting Information [file ADVS-11-2308101-s008.pdf]

ADVANCED SCIENCE
Open Access

Supporting Information

for *Adv. Sci.*, DOI 10.1002/advs.202308101

On-Demand, Contact-Less and Loss-Less Droplet Manipulation via Contact Electrification

*Wei Wang, Hamed Vahabi, Arsalan Taassob, Sreekiran Pillai and Arun Kumar Kota**

Supporting Information

On-demand, Contact-less and Loss-less Droplet Manipulation via Contact Electrification

*Wei Wang[a,b], Hamed Vahabi[c], Arsalan Taassob[a], Sreekiran Pillai[a], Arun Kumar Kota[a,c]**

Section 1. Estimation of the adhesion force F_{adh}

We estimated the adhesion force F_{adh} based on the contact angle hysteresis as:[1]

$$F_{adh} \approx \gamma_{lv} D_{TCL} (\cos\theta_{rec} - \cos\theta_{adv}) \quad \text{(S1)}$$

Here, γ_{lv} is the liquid surface tension, D_{TCL} is the width of the triple phase contact line perpendicular to the droplet sliding direction, and θ_{rec} and θ_{adv} are the receding and advancing contact angles of the liquid droplet on the solid surface. When the contact angle hysteresis is low, the shape of the droplet does not deviate significantly from a spherical cap, and D_{TCL} can be computed as:[2]

$$D_{TCL} = 2\sin\bar{\theta} \left[\frac{3V}{\pi(2 - 3\cos\bar{\theta} - \cos^3\bar{\theta})}\right]^{1/3} \quad \text{(S2)}$$

Here $\bar{\theta}$ is the average contact angle, given as:

$$\cos\bar{\theta} = \frac{(\cos\theta_{adv} + \cos\theta_{rec})}{2} \quad \text{(S3)}$$

For a 50 µl droplet of water (γ_{lv} = 72.1 mN/m) on a Cl-PDMS modified glass surface (D_{TCL} = 5.25 mm, θ_{rec} = 98° and θ_{adv} = 103°), from Equations S1-S3, we estimated the adhesion force $F_{adh} \approx 32$ µN.

Section 2. Estimation of droplet charge q_d

We estimated the droplet charge q_d using a Millikan droplet apparatus, based on a balance between the electrostatic force and the hydrodynamic drag experienced by the droplet. A charged water droplet dispensed into an oil bath in a Millikan droplet apparatus experiences a horizontal electrostatic force:[3]

$$F_{elec} = \frac{q_d V}{d} \quad \text{(S4)}$$

Here, q_d is droplet charge, V is applied voltage between the electrodes and d is distance between the electrodes. The water droplet also experiences a horizontal hydrodynamic drag force:[4, 5]

$$F_{drag} = 4\pi\mu_{oil}R_d U_d \left(\frac{3\lambda+2}{2\lambda+2}\right) \quad \text{(S5)}$$

Here, μ_{oil} is viscosity of the oil, R_d is droplet radius, U_d is horizontal velocity of the droplet, $\lambda = \frac{\mu_d}{\mu_{oil}}$ is viscosity ratio, and μ_d is viscosity of droplet. At equilibrium, $F_{elec} = F_{drag}$. So, from Equations S4 and S5, we get:

$$q_d = \frac{4\pi\mu_{oil}R_d U_d d}{V}\left(\frac{3\lambda+2}{2\lambda+2}\right) \quad \text{(S6)}$$

In our Millikan droplet experiments with a charged water droplet in a silicone oil bath, V = 8000 V, d = 0.074 m, μ_{oil} = 0.019 Pa.s, λ = 0.0526, R_d = 0.00243 m, and U_d = 0.03181 m/s. From Equation S6, we estimated the droplet surface charge density q_d ≈ 0.18 nC.

Section S3. Lossless nature of droplet manipulation

We evaluated the lossless nature of our droplet manipulation technique by measuring the droplet volume, substrate mass and droplet contact angles (advancing and receding) as a function of droplet motion on the Cl-PDMS modified glass surfaces. In each experimental cycle, a 50 μl water droplet was placed on the surface, and it was manipulated back and forth across the slippery surface (transport distance ~10 cm per cycle) using a finger-based PTFE actuator. After every 100 cycles, the droplet image was captured and analyzed with ImageJ to determine the droplet volume; the substrate mass was measured; and separately the advancing and receding contact angles of a new water droplet were measured. If there was liquid loss, with increasing cycles, the droplet volume would have decreased, or the substrate mass would have increased, or the advancing and receding contact angles of water would have changed. However, even after 1000 cycles (total transport distance ~ 100 m), there was no noticeable change in the droplet volume (Figure S1a) or the substrate mass (Figure S1b) or the advancing and receding contact angles of water (Figure S1c). These results confirm that there is no liquid loss associated with our droplet manipulations.

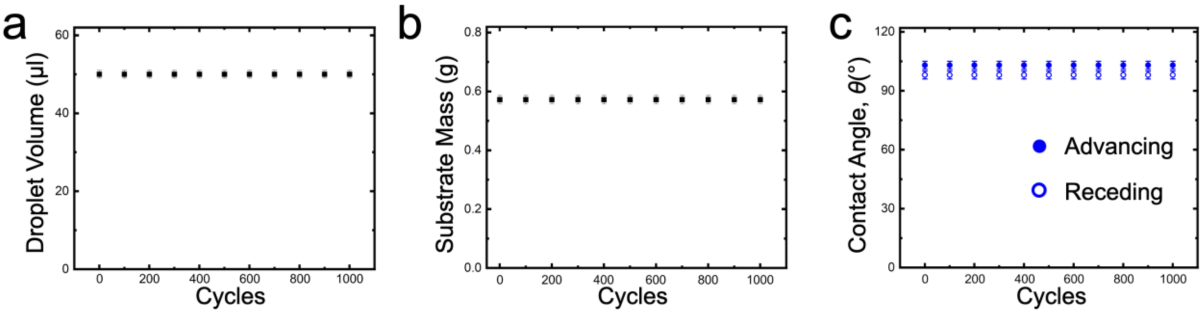

Figure S1. Lossless nature of droplet manipulation. a) Droplet volume, b) substrate mass, and c) water contact angles as a function of droplet motion cycles.

Movie Legends

Movie S1

Attractive droplet manipulation with a negatively charged PTFE actuator.

Movie S2

Repulsive droplet manipulation with a positively charged Nylon actuator.

Movie S3

Uphill motion of a water droplet on OTS modified glass surface inclined at an angle of ~10°.

Movie S4

Transportation and mixing of two miscible droplets – hexadecane (colorless) and toluene (red).

Movie S5

Transportation and mixing of immiscible droplets - water (blue) and hexadecane (colorless).

Movie S6

Demonstration of PTFE wrapped finger as an actuator to transport a droplet of phenol red in water (yellow) along a linear path and mix with sodium hydroxide (colorless) for colorimetric pH indication.

Movie S7

Demonstration of PTFE wrapped finger as an actuator to manipulate a water droplet (blue) along a circular (arbitrary) path.

References

[1] R.J. Good, *J. Adhes. Sci. Technol.* **1992**, 6 (12), 1269-1302.

[2] W. Choi, A. Tuteja, J.M. Mabry, R.E. Cohen, G.H. McKinley, *J. Colloid Interface Sci.* **2009**, 339 (1), 208-216.

[3] A.M. Schoeler, D.N. Josephides, S. Sajjadi, C.D. Lorenz, P. Mesquida, *J. Appl. Phys.* **2013**, 114 (14).

[4] W. Rybczynski, *Bull. Acad. Sci. Series A* **1911**, 40, 33-78.

[5] G.I. Taylor, *Proc. R. Soc. London* **1932**, 138 (834), 41-48.
